# Comparison of rapid immunoassays for rupture of fetal membranes

**DOI:** 10.1186/s12884-017-1311-y

**Published:** 2017-04-26

**Authors:** Irogue Igbinosa, Ferney A. Moore, Cheri Johnson, Jon E. Block

**Affiliations:** 1 Department of Obstetrics & Gynecology, Woman’s Hospital, Louisiana State University Health Sciences, 500 Rue de la vie, Suite 414, Baton Rouge, LA 70817 USA; 2 Woman’s Hospital, 100 Woman’s Way, Baton Rouge, LA 70817 USA; 3 2210 Jackson Street, Suite 401, San Francisco, CA 94115 USA

**Keywords:** ROM plus®, Amnisure®, Premature rupture of membranes, Point of care immunoassay, Insulin-like growth factor binding protein (IGFBP-1), Alpha-fetoprotein (AFP), Placental alpha microglobulin-1 (PAMG-1)

## Abstract

**Background:**

Rupture of membranes (ROM) before the onset of uterine contractions, particularly in pregnancies less than 37 weeks gestational age, is a common diagnostic problem in obstetrical practice. Timely detection of ROM is vital to support gestational age-specific interventions to optimize perinatal outcomes and minimize the risk of serious complications such as preterm delivery, fetal distress and maternal/fetal infections. Rapid bedside immunoassay tests designed to detect amniotic fluid proteins in cervicovaginal fluids have emerged as valuable clinical tools to provide timely ROM diagnosis.

**Methods:**

In this prospective observational study, two commercially-available immunoassay tests (ROM Plus®, AmniSure®) were evaluated concurrently in 111 pregnant women who presented with the chief complaint of ROM. Immunoassay results were compared to clinical parameters for determining ROM via comprehensive, retrospective clinical chart review. Diagnostic performance characteristics were calculated including sensitivity, specificity, positive predictive value (PPV), negative predictive value (NPV) and accuracy.

**Results:**

Overall, diagnostic performance characteristics were robust and similar between ROM Plus® and AmniSure®, respectively: sensitivity (96.4 and 89.3%), specificity (98.8 and 100%), PPV (96.4 and 100%), NPV (98.8% and 96.5) and accuracy (98.2 and 97.3%). For term patients (≥37 weeks gestation), the sensitivities were 93.8 and 81.3% and specificities were 97.1 and 100% for ROM Plus® and AmniSure®, respectively. For preterm patients (<37 weeks gestation), both immunoassay tests provided exact concordance with clinical confirmation of ROM resulting in 100% diagnostic accuracy.

**Conclusions:**

Both rapid immunoassay tests provided similarly excellent diagnostic accuracy for the rapid detection of ROM with only two discrepant results for ROM Plus® and three discrepant results for AmniSure® compared to clinical confirmation. The findings from this study recommend these tests for pregnant women presenting with suspected ROM to guide correct clinical management decisions to improve obstetrical and neonatal outcomes.

**Trial registration:**

ClinicalTrials.gov, NCT02208011 (1 August 2014).

## Background

Rupture of membranes (ROM) has significant clinical implications particularly if it occurs prior to 37 weeks gestation where it is accountable for 20–40% of preterm deliveries [1–3]. In fact, preterm premature ROM (ROM prior to the onset of labor) complicates approximately 2–20% of all pregnancies in the US [4]. Accurate and timely diagnosis of ROM is crucial in guiding appropriate medical management decisions to optimize perinatal outcomes and ameliorate the risk of adverse events [5–8].

When ROM is suspected, traditional diagnostic methods include the use of a sterile speculum examination to visualize leakage of amniotic fluid from the external cervical os or pooling of amniotic fluid in the posterior vaginal fornix, coupled with a microscopic evaluation of a collected specimen for evidence of ferning/crystallization, commonly referred to as the fern test, and pH testing of the fluid with nitrazine test paper [6, 9, 10]. While this approach has remained the standard of care for decades, the results can be equivocal, especially in patients presenting for obstetrical care greater than an hour from the time of suspected ROM [11]. In addition, the sterile speculum exam may be subjective, and it has been shown to have false negatives and false positives which complicate the accurate detection of ROM [6, 12–15]. The “gold standard” to confirm ROM is to inject indigo carmine into the amniotic sac during amniocentesis and then assess whether any blue fluid is visibly leaking from the cervical os or pooling in the vaginal vault [6]. Leakage of the blue stained fluid into the vagina within 20–30 min confirmed by staining of a tampon is regarded as the definitive diagnosis of membrane rupture. As amniocentesis is an invasive procedure with an associated 0.1 – 2% risk of miscarriage, less invasive methods for ROM determination are preferable for routine evaluation.

Several point of care immunoassay tests have been developed, validated and commercialized for the rapid and accurate diagnosis of ROM. These tests detect specific proteins, such as alpha fetoprotein (AFP), found in high concentrations in amniotic fluid but at extremely low background concentrations in cervico-vaginal secretions [16]. The first generation of these tests employed a monoclonal antibody approach focusing on insulin-like growth factor binding protein-1 (IGFBP-1, aka placental protein 12 [PP12]) and placental alpha microglobulin-1 (PAMG-1) [17–21]. More recently, a combined monoclonal/polyclonal antibody immunoassay has been developed to detect two different proteins found in amniotic fluid at high concentrations [22, 23]. This study was undertaken to evaluate the diagnostic accuracy for ROM detection of an original monoclonal antibody immunoassay test compared with a newer monoclonal/polyclonal test.

## Methods

This study compared the diagnostic performance characteristics between two commercially-available, immunochromatographic methods for the detection of ROM: 1) a monoclonal immunoassay that detects the PAMG-1 (AmniSure®, QIAGEN N.V., Netherlands), and 2) a monoclonal/polyclonal immunoassay that detects the presence of IGFBP-1 and AFP (ROM Plus®, Clinical Innovations, Salt Lake City, UT USA).

One hundred eleven (111) pregnant women, ≥ 15 weeks gestation presenting with a complaint of ROM were enrolled in this study between August 2014 and June 2015. Pregnant women with known placenta previa and/or active vaginal bleeding were excluded. This research adhered to Good Clinical Practice (GCP) guidelines and followed the recommendations of the Helsinki Declaration. Each patient provided informed consent before any study-related procedures were performed. This trial was prospectively registered at ClinicalTrials.gov (NCT02208011).

At the time of clinical presentation, both immunoassay tests were performed simultaneously on each patient to determine presence or absence of ROM. The methodological and procedural details of both tests have been detailed previously [18, 23, 24]. In accordance with institutional regulations, both immunoassays were evaluated by authorized and trained personnel in the central hospital laboratory. Pregnant women with discrepant results between the two immunoassay tests underwent a sterile speculum exam (if not previously performed) and sonographic evaluation of amniotic fluid (AFI or 2 × 2 pocket). The patient was considered clinically positive for ROM if either 1) amniotic fluid was seen leaking from the cervical os, or 2) if at least two of the following three clinical signs were present: visual pooling of fluid in the posterior vaginal fornix, positive nitrazine test, and/or microscopic evidence of ferning. A thorough review of the medical records after delivery was undertaken utilizing specific major and minor criteria, prior to establishing the diagnosis of ROM.

The following major criteria were used in the clinician’s review process as highly suggestive of ROM:

- AFI < 7 or no 2 × 2 pocket
- <48 h from initial exam to delivery
- evidence of chorioamnionitis and/or endomyometritis

Clinical confirmation, the final diagnosis of ROM, required the presence of at least two major criteria.

Diagnostic performance characteristics with corresponding 95% confidence intervals (CI) were calculated for each test including sensitivity, specificity, positive predictive value (PPV), negative predictive value (NPV) and accuracy (i.e., overall agreement) [25, 26]. Performance characteristics were computed for the entire patient population as well as separately for subgroups of term (≥37 weeks gestation) and preterm patients (<37 weeks gestation). Additionally, comparable performance characteristics for the sterile speculum exam were computed among the subgroup of pregnant women who underwent this procedure as part of their clinical assessment.

## Results

Of the 111 pregnant women in this study, 51 (46%) were term patients (≥37 weeks gestation) and 60 (54%) were preterm (<37 weeks gestation). For the entire patient population, comparing ROM Plus® results with clinical confirmation per retrospective chart review resulted in overall excellent diagnostic accuracy with a sensitivity of 96.4% (95% CI: 81.7%, 99.9%) and a specificity of 98.8% (95% CI: 93.5%, 100%) (Table 1). The corresponding PPV and NPV for the ROM Plus® were 96.4% (95% CI: 81.7%, 99.9%) and 98.8% (95% CI: 93.5%, 100%), respectively. Only two measurements were discordant between ROM Plus® and clinical confirmation resulting in an overall accuracy of 98.2% (109 of 111).

**Table 1:** Diagnostic performance characteristics in all patients: ROM plus® vs. clinical confirmation

	Clinical confirmation		
ROM Plus	Positive	Negative	Total
Positive	27	1	28
Negative	1	82	83
Total	28	83	111

Sensitivity = 96.4%

Specificity = 98.8%

Comparing the AmniSure® results with clinical confirmation for all patients led to slightly lower sensitivity than ROM Plus® (89.3%, 95% CI: 71.8%, 97.7%) but exact specificity (100%, 95% CI: 95.7%, 100%) (Table 2). The corresponding PPV and NPV for the AmniSure® were 100% (95% CI: 86.3%, 100%) and 96.5% (95% CI: 90.1%, 99.3%), respectively. Three measurements were discordant between AmniSure® and clinical confirmation resulting in an overall accuracy of 97.3% (108 of 111).

**Table 2:** Diagnostic performance characteristics in all patients: Amnisure® vs. clinical confirmation

	Clinical confirmation		
AmniSure	Positive	Negative	Total
Positive	25	0	25
Negative	3	83	86
Total	28	83	111

Sensitivity = 89.3%

Specificity = 100%

The diagnostic performance results for the subgroup of term patients are provided in Tables 3 and 4. Compared to clinical confirmation, the sensitivities were 93.8% (95% CI: 69.8%, 99.8%) and 81.3% (95% CI: 54.4%, 96.0%) and the specificities were 97.1% (95% CI: 85.1%, 99.9%) and 100% (95% CI: 90%, 100%) for ROM Plus® and AmniSure®, respectively. The PPVs were 93.8% (95% CI: 69.8%, 99.8%) and 100% (95% CI: 75.3%, 100%) and the NPVs were 97.1% (95% CI: 85.1%, 99.9%) and 92.1% (95% CI: 78.6%, 98.3%) for ROM Plus® and AmniSure®, respectively. The corresponding accuracy was 96.1% (49 of 51) for ROM Plus® and 94.1% (48 of 51) for AmniSure®.

**Table 3:** Diagnostic performance characteristics in patients ≥ 37 weeks gestation: ROM plus® vs. Clinical confirmation

	Clinical confirmation		
ROM Plus	Positive	Negative	Total
Positive	15	1	16
Negative	1	34	35
Total	16	35	51

Sensitivity = 93.8%

Specificity = 97.1%

**Table 4:** Diagnostic performance characteristics in patients ≥ 37 weeks gestation: AmniSure® vs. clinical confirmation

	Clinical confirmation		
AmniSure	Positive	Negative	Total
Positive	13	0	13
Negative	3	35	38
Total	16	35	51

Sensitivity = 81.3%

Specificity = 100%

For preterm patients, both immunoassay tests provided exact concordance with clinical confirmation of ROM (100%, 60 of 60) (Tables 5 and 6).

**Table 5:** Diagnostic performance characteristics in patients < 37 weeks gestation: ROM plus® vs. clinical confirmation

	Clinical confirmation		
ROM plus	Positive	Negative	Total
Positive	12	0	12
Negative	0	48	48
Total	12	48	60

Sensitivity = 100%

Specificity = 100%

**Table 6:** Diagnostic performance characteristics in patients < 37 weeks gestation: AmniSure® vs. clinical confirmation

	Clinical confirmation		
AmniSure	Positive	Negative	Total
Positive	12	0	12
Negative	0	48	48
Total	12	48	60

Sensitivity = 100%

Specificity = 100%

There were 73 patients (66%) who underwent a sterile speculum exam in this study as a part of their initial evaluation. Compared to clinical confirmation, the sensitivity and specificity of the speculum exam were 72.2% (95% CI: 46.5%, 90.3%) and 96.4% (95% CI: 97.5%, 99.6%) with a PPV and NPV of 86.7% (95% CI: 59.5%, 98.3%) and 91.4% (95% CI: 81.0%, 97.1%), respectively. There were 7 discordant measurements resulting in an accuracy of 90.4%.

## Discussion

Point of care diagnostic testing has revolutionized the medical management of patients with emergent conditions in the acute care setting [27, 28]. Rapid dissemination of results facilitates clinical decision making and improved patient outcomes in settings that often require expedient decisions such as labor and delivery. Traditional diagnostic approaches for ROM such as the sterile speculum exam to identify fluid leakage, nitrazine pH testing and miscroscopic fern analysis all lack consistently sufficient diagnostic accuracy [6, 9, 10, 12–14]. More importantly, ROM detection becomes increasingly ambiguous when more than an hour has elapsed since membrane rupture, underscoring the urgent need for rapid, point of care testing that can be performed easily in various clinical settings [11].

This is the first report of a concurrent evaluation of these two commercially-available immunoassay tests for the diagnosis of ROM. Comparable diagnostic accuracy was achieved by both assays with wide and overlapping confidence intervals. The findings are similar to previously published results showing strong diagnostic accuracy (sensitivity 99%, specificity 91%) for the more newly introduced ROM Plus® assay [22]. Additionally, Rogers et al. [29] also reported similar diagnostic performance characteristics for the ROM Plus® with a sensitivity of 100% and specificity of 94.8% in 75 pregnant patients presenting with suspected ROM.

Both assays provided exact diagnostic accuracy in preterm patients with all measurements in complete agreement between tests and uniformly concordant with clinical confirmation. The risk of complications and worse outcomes increases significantly with lower gestational age making early ROM diagnosis, especially in very early preterm (i.e., ≤ 34 weeks gestation) patients, imperative [30].

Achieving a high level of diagnostic accuracy is particularly important in cases of equivocal membrane rupture as nearly one-quarter of all pregnant women ultimately diagnosed with ROM do not present with overt clinical evidence of ruptured membranes on initial presentation [10]. We found notably lower sensitivity for the sterile speculum exam alone suggesting a diminished ability to correctly identify patients with ROM. Due to their procedural ease of use, these point of care tests can be performed rapidly at the patients’ bedside and provide essential information in understanding the initial clinical scenario of pregnant women with suspected ROM. This diagnostic approach may be very useful in low resource settings where rapid, point of care testing has been shown to be more accurate and cost-effective than a sterile speculum exam [31].

## Conclusions

Both rapid immunoassay tests evaluated in this study provided excellent diagnostic accuracy for the rapid detection of ROM with only two discrepant results for ROM Plus® and three discrepant results for AmniSure® compared to clinical confirmation. Test performance was particularly robust in preterm patients. The findings from this study support a recommendation for these tests in patients presenting with suspected ROM to guide clinical management decisions to improve obstetrical and neonatal outcomes. Future studies with larger patients sample sizes and cost comparison analyses to traditional methods of ROM detection may be helpful as institutions decide on implementation of point of care immunoassays.

## Abbreviations

AFP: Alpha fetoprotein
AFI: Amniotic fluid index
CI: Confidence interval
GCP: Good clinical practice
IGFBP-1: Insulin-like growth factor binding protein-1
NPV: Negative predictive value
PAMG-1: Placental alpha macroglobulin-1
PP12: Placental protein 12
PPV: Positive predictive value
ROM: Rupture of membranes
US: United States
